# Improved Results Over Time With Bridge-to-Lung Transplantation: A 10-Year Experience of a Single High-Volume Center

**DOI:** 10.3389/ti.2025.13944

**Published:** 2025-01-30

**Authors:** Gyungah Kim, Jee Hwan Ahn, Tae Sun Shim, Pil-Je Kang, Geun Dong Lee, Sehoon Choi, Won Kim, Sung-Ho Jung, Dong Kwan Kim, Seung-Il Park, Sang-Bum Hong

**Affiliations:** Asan Medical Center, University of Ulsan College of Medicine, Seoul, Republic of Korea

**Keywords:** lung transplantation, bridge to transplantation, extracorporeal membrane oxygenation, learning curve, ECMO duration

## Abstract

When donor scarcity limits timely lung transplantation (LTx), extracorporeal membrane oxygenation (ECMO) as a bridge to transplantation (BTT) can prolong survival and delay deconditioning until the donor lungs become available. We reviewed 10-year BTT experiences of a single high-volume center, where 99 (59%) were on ECMO BTT among 169 eligible adult LTx cases. Both 28-day and 2-year survivals did not differ between BTT and non-BTT. The BTT data was then divided into two periods, delineated by the most recent 3 years. The clinical outcomes of the earlier period (“Period 1”) and the later period (“Period 2”) were compared, and mortality within 28 days of LTx was significantly lower in Period 2 (n = 1, 1.7%) than in Period 1 (n = 6, 14.6%, *p* < 0.01). Improved survival was observed in the subgroup with BTT duration of 14 days or more. Taken together, more experiences in BTT and improved competence may contribute to better survival after LTx, especially in patients receiving ECMO for 14 days or more.

## Graphical Abstract

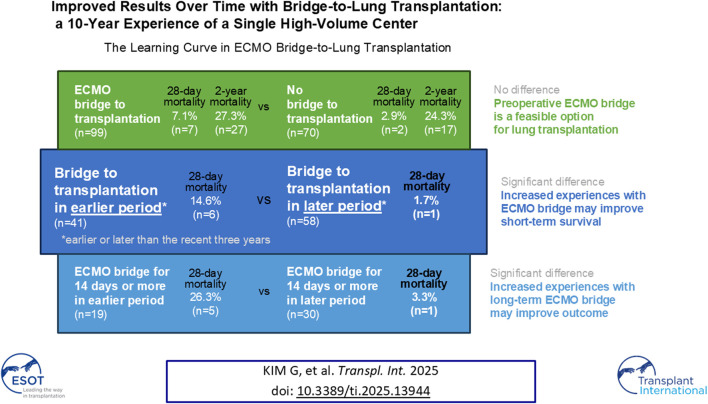

## Introduction

Lung transplantation (LTx) is the treatment option for medically and surgically refractory lung conditions [1]. Despite various efforts and some improvement, waitlist mortality is a problem due to donor shortage [2, 3]. Bridge-to-transplantation (BTT) with extracorporeal membrane oxygenation (ECMO) is used preoperatively to maintain the best possible conditions for LTx by optimizing gas exchange and end-organ perfusion [4, 5]. Rehabilitation is implemented to overcome the deconditioning while on the waitlist [6], and ECMO in awake patients allows active rehabilitation which can improve patients’ outcome [7]. Especially through the coronavirus disease 2019 pandemic, ECMO is increasingly used for severe respiratory failure [8]. However, BTT is frequently associated with complications, ranging from blood clotting-related embolism and serious ischemia of end-organs to bleeding complications and catheter site problems [9–12].

Clinical outcomes of BTT vary among centers, with 1-year survivals ranging from 29% to 93% [13]. One of the factors associated with the inter-center discrepancies is the annual number of BTT at the LTx center. A retrospective review of all LTx recipients in the United Network for Organ Sharing dataset from May 2005 to June 2011 revealed that survival of patients with high risk (high lung allocation scores, requiring mechanical ventilation (MV) or ECMO support) was better in high-volume centers compared to in low-volume centers [14]. Accumulated know-hows in BTT have been demonstrated to bring better clinical outcomes for the patients undergoing LTx [15, 16].

The learning curve of BTT in LTx with a large number of BTT cases has yet to be studied. We aimed to investigate whether BTT experiences over time in a high-volume center result in improved clinical outcomes. We hypothesized that accumulated experiences in BTT improve the survival of LTx patients. We further explored the factors associated with improved survival and the subgroup with the most improvement.

## Materials and Methods

### Study Population

The data was retrieved retrospectively from patients over 19 years of age (legal age for adulthood in Korea) who received LTx at Asan Medical Center in Seoul, Republic of Korea during 2008 and 2021. Patients with liver-lung simultaneous transplantations were excluded. Patients were followed until death or December 2023. To estimate clinical severity, SAPS II was used in this study because it was validated in medical ICU patients and patients with respiratory failure on ECMO and employed in studies of BTT LTx patients [17–19].

LTx was achieved solely through strictly regulated process by the relevant legislation, and all organs used for transplantation were freely given with written informed consent by donors or family members through the government agency, the Korean Network for Organ Sharing. The study protocol was approved by the institutional review board of Asan Medical Center (approval number 2020–0209) and the requirement for informed consent was waived because of the retrospective nature of the study and the use of anonymized clinical data.

### Study Design

LTx cases were divided into BTT and non-BTT cases and compared. BTT group was then divided into two period groups based on whether LTx was performed before (“Period 1”) or within (“Period 2”) the most recent 3 years (2019–2021) during the study period. The two periods were further categorized into subgroups according to the duration of BTT (short-term vs long-term), with the reference duration of 14 days based on previous studies [20, 21]. Clinical outcomes were compared.

### Outcomes

The primary outcomes were 28-day and 2-year mortality, and the secondary outcomes were hospital and intensive care unit (ICU) lengths of stay, primary graft dysfunction (PGD), postoperative MV duration and MV-free days, and requirement for postoperative tracheostomy.

### Clinical Strategies

#### LTx Protocol

Patients with end-stage lung diseases except lung cancer were considered for LTx and selected according to the recommendations of the International Society for Heart and Lung Transplantation [22]. After the confirmation of the suitability for LTx by an institutional multidisciplinary committee comprised of pulmonologists, intensivists, cardiothoracic surgeons, infectious disease specialists, anesthesiologists, and radiologists, the candidate was listed through the Korean Network for Organ Sharing for donor lung allocation according to the urgency status, which gives the most urgency priority (status 0) only to the patients requiring MV or ECMO [23, 24]. The committee re-evaluate the condition of both donor and recipient at the time of donor lung availability to decide to proceed LTx, meticulously checking for the contraindications for LTx and the risk factors for poor post-transplant outcomes such as untreatable major organ dysfunction, uncorrectable bleeding diathesis, and limited functional status with poor rehabilitation potential [22]. Bilateral total lung transplantation rather than single or lobar lung transplantation and standard-criteria donor lungs rather than extended-criteria donor lungs were utilized as much as possible. Cardiopulmonary support during transplant surgery consisted of central veno-arterial (V-A) ECMO or cardiopulmonary bypass (CPB) with most patients weaned from the support at the end of LTx, although V-A or veno-venous (V-V) ECMO was applied postoperatively according to the recipient’s conditions, which continued until recovery or death.

#### ECMO Protocol

ECMO as BTT was managed as recommended by the Extracorporeal Life Support Organization [25]. ECMO BTT was considered in LTx candidates with refractory hypoxemia, hypercarbia, or right heart failure despite optimal medical treatment. Patients on BTT were tracheostomized or extubated within a few days from the ECMO support to maximize mobilization, unless LTx was proceeded before tracheostomy or extubation. Patients with BTT were mobilized as soon as possible to preserve the skeletal muscle mass. BTT was not applied to patients who did not require MV or who were capable of rehabilitation without BTT despite the application of MV. Patients ineligible for LTx were not considered for BTT, and factors such as old age (older than 65 years of age), limitations in vascular access, uncontrolled sepsis, coagulopathy, and prolonged MV were also considered before starting ECMO.

Cannulation was performed and configuration was carefully selected based on individual patient conditions [5]. Intensive care physicians executed comprehensive evaluation of the cardiac function including right ventricular function by performing cardiac ultrasound, checking cardiac enzymes and brain natriuretic peptide, and assessing hemodynamic stability. Comprehensive echocardiography was performed by cardiologists if necessary. Intensivist routinely re-evaluate cardiac functions of bridged patients. The ECMO configuration was changed based on the clinical evaluation. V-V ECMO was primarily applied to patients with hypoxemic respiratory failure without hemodynamic instability, and V-A ECMO was applied to patients requiring hemodynamic support. Veno-arteriovenous (V-AV) ECMO was considered in differential hypoxia. Configuration changes to V-A, V-AV, or right ventricular assist device with an oxygenator (OxyRVAD) were considered in patients developing right ventricular dysfunction, as previously described [26]. To briefly describe OxyRVAD, the main pulmonary artery was approached by left anterior mini-thoracotomy and then a graft was anastomosed to the main pulmonary artery. Next, an arterial reinfusion cannula was connected to the graft, followed by the initiation of the RVAD. In this study, configuration changes only include changes during the preoperative BTT. Efforts were made to awaken all patients to participate in maximal rehabilitation while on BTT support, and central ECMO such as OxyRVAD was considered for further engagement in physical rehabilitation [27, 28].

The QUADROX PLS System (Maquet Cardiopulmonary AG, Rastatt, Germany) and the CAPIOX EBS System (Terumo Cardiovascular Systems Corporation, Tokyo, Japan) were used, each with its own oxygenator, pump, and console. Unfractionated heparin was predominantly used as intravenous anticoagulation during BTT, and argatroban was alternatively used for confirmed or suspected heparin-induced thrombocytopenia [29], with the dose titrated to achieve a target activated partial thromboplastin time of 40–60 s. A bolus of unfractionated heparin (50–70 units/kg) was infused at the start of ECMO support and usually 800 units/hour of unfractionated heparin was initiated, and then dosage was adjusted based on the activated partial thromboplastin time. Complications were monitored, and bleeding complications included major bleeding requiring surgical or radiological interventions as well as minor bleeding requiring close monitoring. Leg ischemia complication was defined as requiring decannulation, fasciotomy, amputation, or new distal perfusion cannulation.

### Statistical Analysis

Categorical variables were presented as frequencies and proportions in percent and continuous variables were presented as medians and interquartile ranges. For intergroup comparison, the Student’s *t* test or the Mann-Whitney U test were used for continuous variables, and the Chi-square test or the Fisher exact test were used for categorical variables. Survival curves were estimated using the Kaplan-Meier method and the log-rank test was performed for intergroup comparison. Univariate analysis was performed to identify risk factors for 28-day mortality, and a Cox proportional hazard regression model was used for multivariable analysis to assess the relationship between the independent variable and the post-transplantation mortality with *p* < 0.10 for inclusion of variables. The hazard ratios (HR) and 95% confidence intervals (CI) were presented. All statistical analyses were two-sided, and the level of significance was set to type I error rate of 0.05. Statistical analysis was performed using R version 3.4.4 (R Foundation for Statistical Computing, Vienna, Austria).

## Results

### Study Population

**FIGURE 1:**
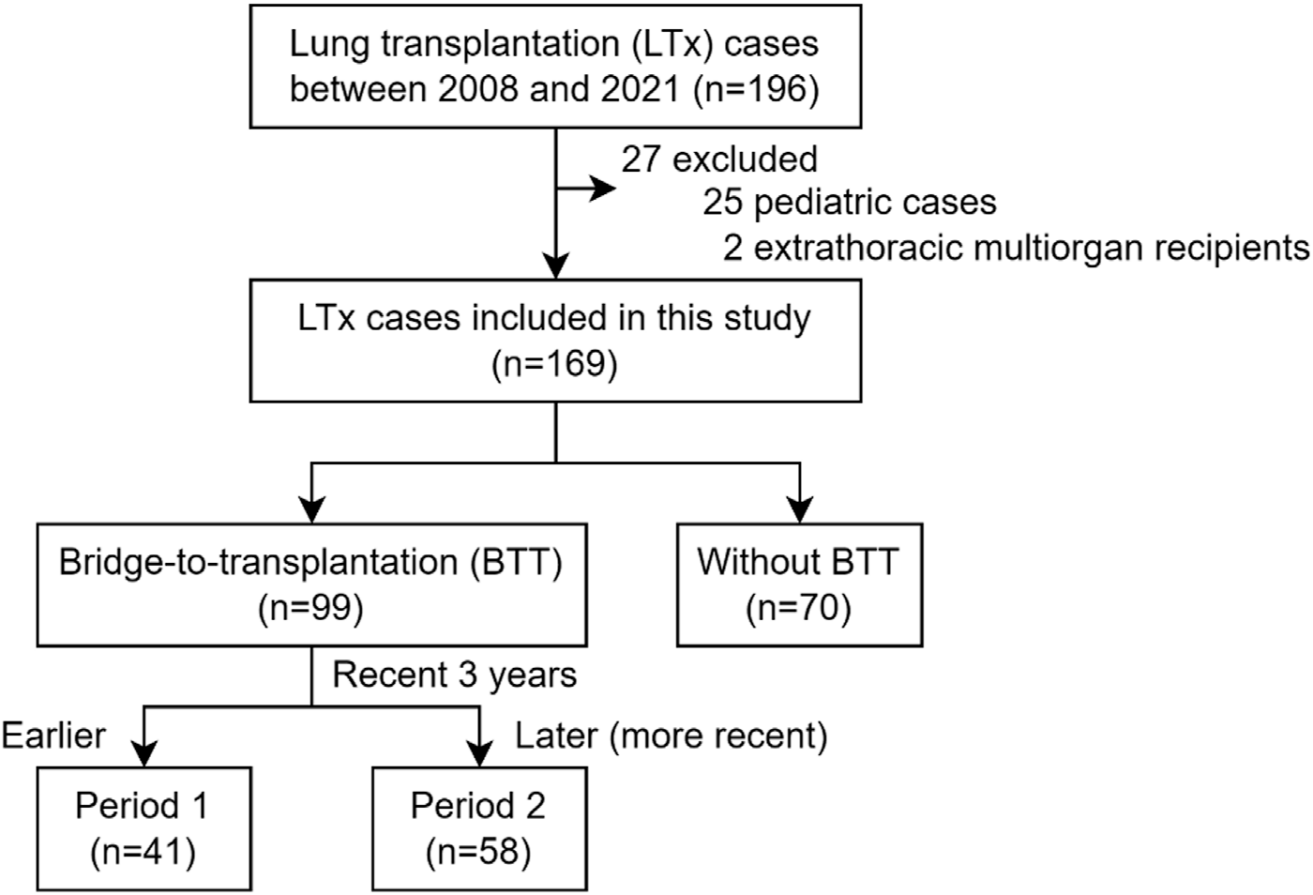
Study population.

From 2008 to 2021, 196 cases underwent LTx, and 169 cases were included in this study after excluding 25 pediatric cases and 2 cases receiving simultaneous liver transplantation (Figure 1). Based on the application of extracorporeal life support before LTx, 99 cases (58.6%) were bridged on ECMO (BTT group), and 70 cases (41.4%) were not on BTT (non-BTT group). The BTT group was further divided into earlier (Period 1, n = 41) and later (Period 2, n = 58) period groups, delineated by the most recent 3 years within the study period.

### Basic Characteristics

The number of total LTx cases at our center increased over time since the beginning of LTx in 2008. While 77 cases received LTx during the first 10 years (2008–2018), 92 cases underwent LTx during the subsequent 3 years between 2019 and 2021 (Supplementary Figure S1A). The median age of the study population was 57 [44–63], and 63.3% (n = 107) were males. More than half (n = 112, 66.3%) were diagnosed with interstitial lung diseases (ILD) among whom idiopathic pulmonary fibrosis was the most common (n = 59), and 121 (71.6%) were Status 0 (i.e., on MV or ECMO) at the time of transplantation. There was no loss in follow-up during the 2-year post-transplant period for the investigated outcomes.

Our center began performing BTT in 2011. The number of BTT cases also rapidly expanded around the beginning of 2019, and cases per year exceeded 10 since then. The proportion of BTT has also increased over the years, and 75.0% were bridged on ECMO in 2021 (Supplementary Figure S1B). At the time of transplantation, 32 cases (32.3%) were on central ECMO, and 67 cases (67.7%) were on peripheral ECMO.

### Comparison Between Earlier (Period 1) and Later (Period 2) Periods

The BTT group was further divided into earlier (Period 1, n = 41) and later (Period 2, n = 58) groups based on whether LTx was performed during the recent 3 years or earlier.

#### Epidemiologic and Clinical Characteristics

Compositions of the patient from two period groups were generally homogeneous, with unvaried sex, body mass index (BMI), diagnosis, preoperative hospital stay, BTT duration, and total hospital stay (Table 1). The median ages were 55 [42–62] years for Period 1 and 60 [54–64] years for Period 2 (*p* = 0.04). A substantial difference was noted in intraoperative circulatory support, as V-A ECMO was introduced and mostly replaced CPB (n = 3, 7.3% in Period 1 vs n = 57, 98.3% in Period 2, *p* < 0.01). SAPS II at the time of LTx was significantly higher in Period 1 [35 (31–45)] than in Period 2 [29 (26–35)] (*p* < 0.01).

**TABLE 1:** Basic characteristics of BTT group in Period 1 and Period 2.

		Period 1 (n = 41)	Period 2 (n = 58)	*p*-value
Age		55 [42–62]	60 [54–64]	0.04
Sex	Female	15 (36.6%)	25 (43.1%)	0.66
	Male	26 (63.3%)	33 (56.9%)	
BMI		23.1 [20.3–25.3]	22.5 [19.8–25.3]	0.68
Diagnosis	ILD	30 (73.2%)	41 (70.7%)	0.66
	Bronchiolitis obliterans	2 (4.9%)	2 (3.4%)	
	Acute respiratory distress syndrome	6 (14.6%)	13 (22.4%)	
	Pulmonary hypertension	3 (7.3%)	2 (3.4%)	
Hospital days to LTx (days)		29 [14–41]	34 [17–48]	0.33
BTT duration (days)		13 [8–17]	15 [4–26]	0.57
Long term BTT	≥14 days	19 (46.3%)	30 (51.7%)	0.75
Configuration change		15 (36.6%)	29 (50.0%)	0.26
Configuration at LTx	V-V	29 (70.7%)	30 (51.7%)	<0.01
	V-A	10 (24.4%)	1 (1.7%)	
	V-AV	1 (2.4%)	2 (3.4%)	
	OxyRVAD	1 (2.4%)	25 (43.1%)	
Intraoperative support	CPB	38 (92.7%)	1 (1.4%)	<0.01
	V-A ECMO	3 (7.3%)	57 (98.3%)	
Preoperative rehabilitation	Rehabilitation	28 (75.6%)	44 (75.9%)	1.00
	Immobile	10 (24.4%)	14 (24.1%)	
Tracheostomy		10 (24.4%)	15 (25.9%)	0.26
Renal replacement therapy during BTT		5 (12.2%)	4 (6.9%)	0.58
ECMO complications	Total	15 (36.6%)	27 (46.6%)	0.43
	Pump clot	9 (22.0%)	6 (10.3%)	0.19
	Catheter site	7 (17.1%)	6 (10.3%)	0.50
	Bleeding	13 (31.7%)	26 (44.8%)	0.27
SAPS II		35 [31–45]	29 [26–35]	<0.01

#### Clinical Outcomes

**FIGURE 2:**
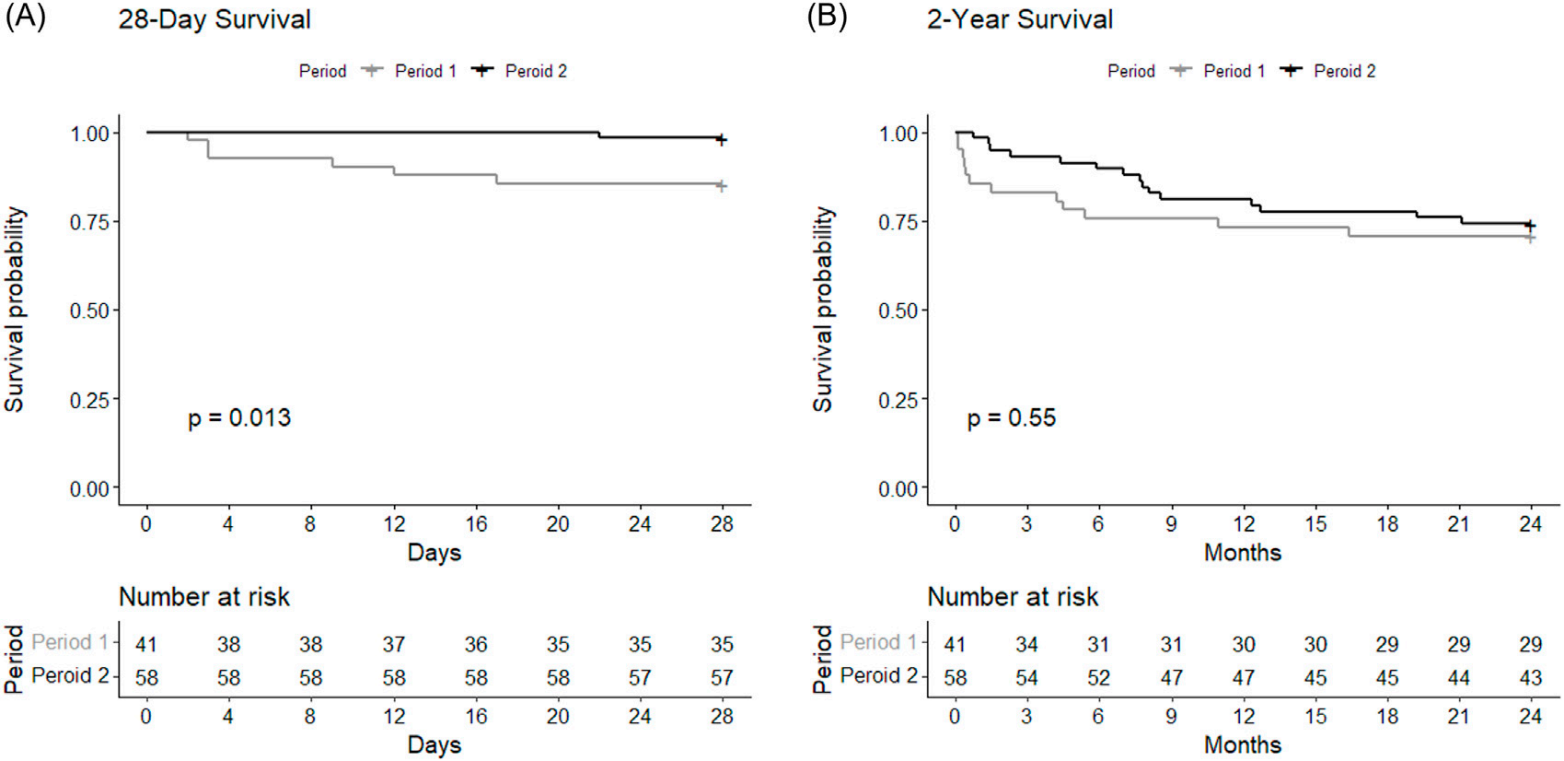
Survival curves showing **(A)** 28-day and **(B)** 2-year postoperative survivals of Period 1 and Period 2 among BTT group. The curves were compared between the periods using log-rank test.

Comparison of clinical outcomes between Period 1 and Period 2 among BTT group is shown in Table 2. Notably, 28-day mortality was significantly higher (HR = 0.11, 95% CI = 0.01–0.91, *p* = 0.01) in Period 1 (n = 6, 14.6%) compared to Period 2 (n = 1, 1.7%) (Figure 2A). No significant difference was observed in 2-year mortality (HR = 0.79, 95% CI = 0.37–1.7, *p* = 0.55) (Figure 2B), PGD, hospital or ICU lengths of stay, postoperative MV duration and MV-free days. Postoperative tracheostomy was required less frequently during Period 2 (n = 6, 10.3%) compared to Period 1 (n = 23, 60.5%, *p* < 0.01). Postoperative ECMO was required in 7.3% (n = 3) of Period 1% and 15.5% (n = 9) of Period 2 (*p* = 0.36).

**TABLE 2:** Clinical outcomes of BTT group in Period 1 and Period 2.

	Period 1 (n = 41)	Period 2 (n = 58)	*p*-value
28-day mortality	6 (14.6%)	1 (1.7%)	0.04
2-year mortality	12 (29.3%)	15 (25.9%)	0.88
Hospital length of stay (days)	105 [58–146]	147 [81–197]	0.07
ICU length of stay (days)	40 [25–54]	50 [29–82]	0.10
PGD			0.30
Grade 1	6 (14.6%)	7 (12.1%)	
Grade 2	10 (24.4%)	7 (12.1%)	
Grade 3	6 (14.6%)	7 (12.1%)	
Postoperative MV duration (days)	11 [6–18]	13 [6–27]	0.37
Postoperative MV-free days (/30 days)	15 [0–24]	18 [3–24]	0.65
Postoperative tracheostomy	23 (60.5%)	6 (10.3%)	<0.01

Univariate analysis on 28-day mortality identified factors associated with surviving population as age at LTx, preoperative rehabilitation, ECMO site complications, intraoperative support, and SAPS II (Supplementary Figure S2). Multivariable analysis using logistic regression did not reveal any statistically significant factors associated with 28-day mortality (Supplementary Figure S3).

#### Subgroups Based on BTT Duration

**FIGURE 3:**
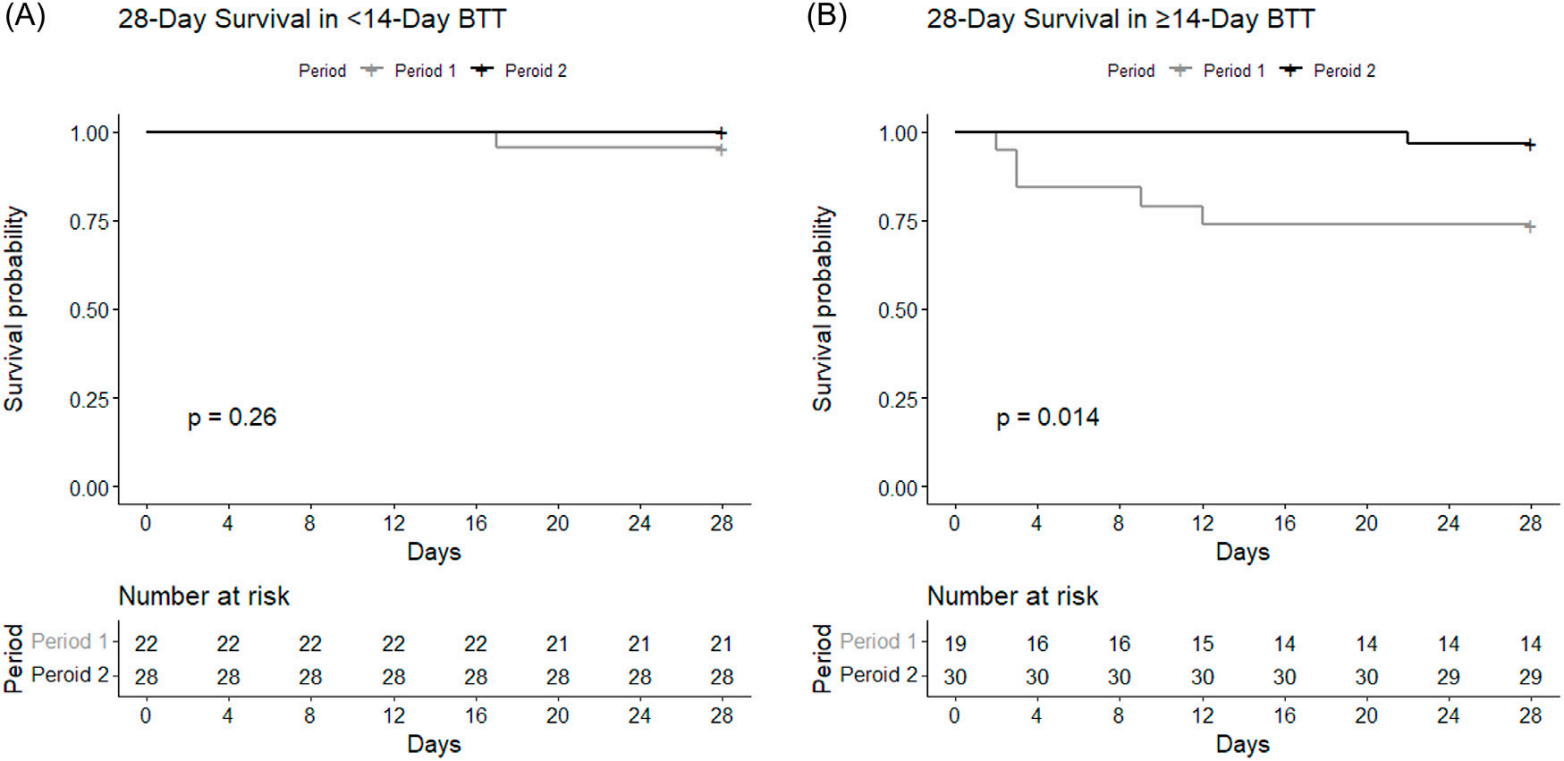
Survival curves depicting 28-day survival according to the duration of BTT. Survival rates of subgroups within BTT group with BTT durations of ECMO for **(A)** less than 14 days and **(B)** 14 days or longer were each compared between Period 1 and Period 2.

The BTT group was further divided according to the duration of BTT (short-term vs long-term) and compared between Period 1 and Period 2 (Figure 3). The short-term BTT group (bridged for less than 14 days) showed similar 28-day survival rates between Period 1 (n = 21, 95.5%) and Period 2 (n = 28, 100.0%, *p* = 0.26) (Figure 3A). Long-term BTT group (bridged for 14 days or more) showed significantly improved 28-day survival in Period 2 (n = 29, 96.7%) compared to Period 1 (n = 14, 73.7%, *p* = 0.01) (Figure 3B). In the short-term BTT subgroup, 68.0% (n = 34) were able to participate in rehabilitation, compared to 83.7% (n = 41) in the long-term BTT subgroup (*p* = 0.11).

#### Configuration Change

Initial ECMO configuration was mostly V-V in both periods (n = 35, 85.4% in Period 1 vs n = 50, 86.2% in Period 2, *p* = 0.21). The frequency of configuration change was similar between Period 1 (n = 15, 36.6%) and Period 2 (n = 29, 50.0%, *p* = 0.26) (Table 1). The final configuration at the time of LTx was also mostly V-V (n = 29, 70.7% in Period 1 vs n = 30, 51.7% in Period 2), and OxyRVAD was more frequently utilized during Period 2 (n = 25, 43.1%) compared to Period 1 (n = 1, 2.4%, *p* < 0.01). The 28-day mortality rates for V-V ECMO (n = 4, 6.8%), V-A and V-AV ECMO (n = 2, 14.3%), and OxyRVAD (n = 1, 3.8%) did not differ (*p* = 0.47).

### Comparison Between BTT and Non-BTT

Compared with non-BTT group (n = 70), BTT group (n = 99) was associated with a higher BMI at operation [22.6 (19.9–25.3) vs. 21.0 (17.8–24.2) kg/m^2^, *p* = 0.01], and a higher proportion of acute respiratory distress syndrome (n = 20, 20.2% vs. n = 5, 7.1%, *p* < 0.01) (Supplementary Table S1). BTT group included more Status 0 (n = 99, 100.0% vs. n = 22, 31.4%, *p* < 0.01) and more patients on MV (n = 99, 100.0% vs n = 22, 31.4%, *p* < 0.01). BTT group showed longer hospital days to transplantation [32 (16–43) vs. 0 (0–16) days, *p* < 0.01] and a higher SAPS II at LTx [33 (28–36) vs. 12 (10–22), *p* < 0.01] than non-BTT group.

Initial ECMO configuration was predominantly V-V (n = 85, 85.9%), followed by V-A (n = 9, 9.1%), V-AV (n = 2, 2.0%), and OxyRVAD (n = 2, 2.0%). The configuration was changed before LTx in 23 cases (23.2%), and the configuration at time of transplantation was mostly V-V (n = 59, 59.6%), followed by OxyRVAD (n = 26, 26.3%), V-A (n = 11, 11.1%), and V-AV (n = 3, 3.0%). No statistical difference was found in clinical outcomes between those with and without configuration changes.

Post-transplantation mortality between BTT and non-BTT cases did not significantly differ at 28 days (n = 7, 7.1% vs. n = 2, 2.9%, *p* = 0.31) or 2 years (n = 27, 27.3% vs n = 17, 24.3%, *p* = 0.80) (Supplementary Table S2). PGD was also similar between the two groups. BTT group showed longer hospital and ICU lengths of stay and postoperative MV duration. Postoperative ECMO was applied in 12.1% (n = 12) of BTT and 5.7% (n = 4) of non-BTT groups (*p* = 0.26). Similar proportions required tracheostomy postoperatively between BTT and non-BTT groups. Survival analysis showed similar 28-day (HR = 2.57, 95% CI = 0.47–26.2, *p* = 0.31) and 2-year (HR = 1.17, 95% CI = 0.64–2.14, *p* = 0.62) mortality rates between the non-BTT and BTT groups. Re-transplantation did not differ between BTT and non-BTT groups, which occurred in one case from each group (1.0% vs. 1.4%, *p* = 1.00), and the reasons for re-transplantation were chronic lung allograft dysfunction for the BTT case and acute rejection for the non-BTT case. One case of leg ischemia occurred among those with peripheral arterial cannulas.

## Discussion

This high-volume single-center retrospective observational study showed BTT in later period was associated with better 28-day survival compared to earlier period. The improvement in 28-day survival was especially apparent in preoperative BTT for 14 days or longer. Also, 2-year mortality did not differ between BTT and non-BTT undergoing LTx, suggesting BTT is a feasible option for patients with end-stage lung diseases.

BTT outcomes are different among centers. For example, a systematic review showed 1-year survivals ranging from 29% to 93% [13]. Some studies previously showed compromised overall mortality for BTT [14, 30]. A report of 26 ECMO patients showed 27% survived until hospital discharge after LTx [31]. A recent study of 40,866 LTx patients showed worse 2-year survival for ECMO patients (53.8%) than for non-ECMO patients (61.8%) [32]. In this regard, preoperative ECMO was previously considered a contraindication to LTx due to unfavorable outcomes [33, 34]. Other studies, however, showed no difference in survival regarding BTT. A report of 71 patients with intention of BTT showed 89% survived through LTx, and 1-year, 3-year, and 5-year survival was 66%, 58%, and 48%, respectively [5]. Likewise, studies showed similar overall survival between BTT and non-BTT [16, 33]. Consistent with these latter studies, our study showed overall survival at 2 years of BTT cases similar to that of non-BTT cases. Notably, the 2-year survival for the BTT group in this study was 72.7% (n = 72, Supplementary Table S2), higher than most of aforementioned studies.

High-volume LTx centers may have better outcomes with BTT possibly due to protocolized institutional support that must be established over time through accumulation of clinical experiences. In one study, high-volume centers with more than 30 total LTx cases per year showed improved survival for BTT patients [14]. Another study of the United Network for Organ Sharing database investigated 342 BTT cases and showed better 1-year survival in high-volume centers, with “high-volume” defined as more than 15 BTT cases during the 15-year period [15]. In our center, the proportion of BTT (58.6%) and the number of BTT cases (99 cases in a 14-year duration) were much higher than those in high-volume centers of previous studies. The following factors may explain the increased use of BTT in our center (Supplementary Figure S1). First, the increased experience with BTT could lead to competence, which allowed our lung transplantation team to accommodate more BTT cases. Second, the globally increased experiences and advances in ECMO through the pandemics justified the choice of BTT [8, 35]. More literature reported benefits of ECMO including awake ECMO bridging in lung transplantation candidates [7, 36].

The donor lung allocation may explain the exceptionally high proportion of BTT in our center. Donor shortage is a problem especially in Korea because Korea only accepts donation after neurologic death and families are often unwilling to donate perhaps due to Korea’s conservative culture. Moreover, donor lungs are vulnerable to damage, limiting their availability [37]. On average, there were 489 donors per year in Korea over the past 5 years, and only 159 out of 489 (32.5%) donor lungs were used for lung transplantation according to Korea Organ Donation Agency. The high proportion of BTT is also likely influenced by the urgency-based donor lung allocation system in Korea, which gives highest priority to Status 0 patients on invasive MV or ECMO [24]. Status 0 is responsible for 64% (n = 104) of annual lung transplantation (n = 162) in Korea according to the Center for Korean Network for Organ Sharing. This suggests that Korean patients with end-stage lung diseases on the waitlist often have to wait until they cannot go further without MV or ECMO before they are able to receive lung transplantation. Furthermore, our center is a tertiary referral center and patients with the most severe diseases are referred nationwide. These factors may have contributed to the high proportion of BTT in our center.

As more experiences lead to competence, we hypothesized that later period in a center’s LTx history with acquired expertise would result in better clinical outcomes compared to earlier period. This was demonstrated in a recent study with the United Network for Organ Sharing database [32]. Learning curves in ECMO have been observed in previous studies, as centers experienced with more annual ECMO cases have better survival rates [38, 39]. Similarly, we found survival at 28 days was higher in Period 2 compared to Period 1 (Figure 2A), which might imply that the precedent 10-year period was a steppingstone for improvement. Experiences with around 40 BTT cases in our center may have equipped our team to maintain stable physiological states of the waitlist patients on ECMO, resulting in lower SAPS II at the time of LTx during Period 2 (Table 1).

We attempted to identify the factors associated with 28-day mortality following LTx. The univariate analysis identified age, the use of V-A ECMO or CPB for intraoperative support, ECMO site complications, operation of rehabilitation program, and SAPS II as factors associated with 28-day mortality (Supplementary Figure S2). Some of these factors have been identified as clinically important in previous reports [6, 25, 40]. However, multivariable analysis using a Cox proportional hazard regression model showed none of the factors associated with 28-day mortality. These discrepancies may be explained by the small number of mortality cases (n = 7), which was not sufficient to yield statistically significant results. Also, the factors identified in the univariate analysis may not be independent determinants of 28-day mortality. Further studies may be required to determine the factors associated with 28-day mortality in BTT.

BTT cases were further divided into subgroups based on the duration of preoperative ECMO, and the earlier and later period groups showed differences in 28-day mortality only in the subgroup with BTT for 14 days or longer (Figure 3). Previous studies showed BTT for longer than 14 days was associated with poorer outcome [20, 21]. In this study, the improved 28-day mortality rate in the later period was mostly attributable to improvement in those with BTT for longer than 14 days. Increased duration of ECMO exposes patients to more complications which can make clinical management difficult [12]. Only the 28-day survival, not the 2-year survival, showed differences, which may suggest that increased BTT experiences particularly improves more immediate postoperative management, while long-term outcomes are affected by many different factors that are not entirely explained by the learning curve alone. Future studies should aim to improve long-term outcomes as well as short-term outcomes.

OxyRVAD was more frequently used in Period 2. We previously showed that OxyRVAD should be considered to support right heart dysfunction and to facilitate preoperative rehabilitation [26–28, 41]. In our cohort, the 28-day mortality with OxyRVAD (n = 1, 3.8%) was not higher compared to V-A or V-AV ECMO (n = 2, 14.3%), suggesting OxyRVAD is noninferior to other configurations. Our team recently showed proper configuration change from V-V ECMO in patients with increasing lactate levels and vasoactive inotropic scores may prevent clinical deterioration [26]. OxyRVAD is an option for patients on V-V ECMO developing right heart failure, stabilizing hemodynamics and enabling active rehabilitation to maintain the best fit for transplantation [41]. More investigations are needed to clarify the contributions of OxyRVAD during BTT.

This study examined changes in BTT outcomes over time in a large-volume single center with a high survival rate. The strength of our study is the inclusion of a large number of long-standing BTT cases compared to previous studies, as our cohort involved a large proportion of BTT (n = 99, 58.6%) and a long median duration of BTT (around 15 days). This study attempts to investigate factors and subgroups associated with improvement over time. There are limitations. Although the number of BTT cases was relatively large, only 7 cases died within 28 days past LTx, which makes further statistical analysis difficult to perform due to the small sample size. The retrospective nature of the study limits the interpretation of the results. This study shares experiences of a single center and the results cannot be generalized.

In conclusion, accumulation of experiences over time is associated with improved 28-day mortality in BTT for LTx, especially in BTT for 14 days or longer. BTT is a feasible option for LTx, with similar 28-day and 2-year survival rates compared to non-BTT.

## Data Availability

The raw data supporting the conclusions of this article will be made available by the authors, without undue reservation.

## Abbreviations

BMI, Body mass index; BTT, Bridge-to-transplantation; CI, Confidence interval; CPB, Cardiopulmonary bypass; ECMO, Extracorporeal membrane oxygenation; V-V, Veno-venous; V-A, Veno-arterial; V-AV, Veno-arteriovenous; HR, Hazard ratio; ILD, Interstitial lung disease; MV, Mechanical ventilation; ICU, Intensive care unit; LTx, Lung transplantation; OxyRVAD, Right ventricular assist device with an oxygenator; PGD, Primary graft dysfunction; SAPS II, Simplified Acute Physiology Score II.
