# Supplementary material for: Improved Results Over Time With Bridge-to-Lung Transplantation: A 10-Year Experience of a Single High-Volume Center
Source: Transpl Int. 2025 Jan 30;38:13944. doi: 10.3389/ti.2025.13944 (PMC11821422; doi:10.3389/ti.2025.13944)
Supplement: Supplementary file 1 [file DataSheet1.docx]

**Supplementary Information**

Contents

*Tables*

Supplementary Table 1. Basic characteristics of BTT and non-BTT groups.

Supplementary Table 2. Outcomes of BTT and non-BTT groups.

*Figures*

Supplementary Figure 1. BTT over time among LTx cases included in this study.

Supplementary Figure 2. Univariate analysis on risks for 28-day mortality.

Supplementary Figure 3. Multivariable analysis on risks for 28-day mortality.

Supplementary Table 1. Basic characteristics of BTT and non-BTT groups.

|  |  | **BTT**  **(n=99)** | **Non-BTT**  **(n=70)** | ***p*-value** |
| --- | --- | --- | --- | --- |
| **Age** |  | 58 [47-63] | 55 [43-61] | 0.07 |
| **Sex** | Female | 40 (40.4%) | 22 (31.4%) | 0.30 |
|  | Male | 59 (59.6%) | 48 (68.6%) |  |
| **BMI** |  | 22.6 [19.9-25.3] | 21.0 [17.8-24.2] | **0.01** |
| **Diagnosis** | ILD | 71 (71.7%) | 41 (58.6%) | **<0.01** |
|  | BO | 4 (4.0%) | 5 (7.1%) |  |
|  | ARDS | 20 (20.2%) | 5 (7.1%) |  |
|  | COPD | 0 (0.0%) | 6 (8.6%) |  |
|  | BE | 0 (0.0%) | 6 (8.6%) |  |
|  | PHTN | 4 (4.0%) | 7 (10.0%) |  |
| **Urgency status** | Status 0 | 99 (100.0%) | 22 (31.4%) | **<0.01** |
|  | Status 1 | 0 (0.0%) | 42 (60.0%) |  |
|  | >Status 2 | 0 (0.0%) | 6 (8.6%) |  |
| **Preoperative MV** | On MV | 99 (100.0%) | 22 (31.4%) | **<0.01** |
| MV duration (days) |  | 15 [8-31] | 0 [0-13] | **<0.01** |
| **HD to LTx (days)** |  | 32 [16-43] | 0 [0-16] | **<0.01** |
| **Rehabilitation** | Rehab | 24 (24.2%) | 9 (12.9%) | 0.10 |
| **PaO_2_/FiO_2_ ratio** |  | 75.0 [58.1-97.2] | 205.8 [171.7-282.9] |  |
| **SAPS II** |  | 33 [28-36] | 12 [10-22] | **<0.01** |

BTT; bridge-to-transplantation, BMI; body-mass index, ILD; interstitial lung disease, BO; bronchiolitis obliterans, ARDS; acute respiratory distress syndrome, COPD; chronic obstructive pulmonary disease, BE; bronchiectasis, PHTN; pulmonary hypertension, MV; mechanical ventilation, HD; hospital days, LTx; lung transplantation, SAPS II; Simplified Acute Physiology Score II

Supplementary Table 2. Outcomes of BTT and non-BTT groups.

|  | **BTT**  **(n=99)** | **Non-BTT**  **(n=70)** | ***p*-value** |
| --- | --- | --- | --- |
| 28-day mortality | 7 (7.1%) | 2 (2.9%) | 0.31 |
| 2-year mortality | 27 (27.3%) | 17 (24.3%) | 0.80 |
| Hospital length of stay (days) | 117 [65-196] | 59 [31-84] | **<0.01** |
| ICU length of stay (days) | 44 [26-71] | 16 [10-31] | **<0.01** |
| Postoperative ICU stay (days) | 22 [13-36] | 15 [10-23] | **<0.01** |
| Postoperative MV duration (days) | 11 [6-23] | 6 [4-13] | **<0.01** |
| Postoperative MV-free days (/30 days) | 16 [0-24] | 24 [18-26] | **<0.01** |
| Postoperative tracheostomy | 29 (30.2%) | 14 (20.6%) | 0.23 |

BTT; bridge-to-transplantation, ICU; intensive care unit, MV; mechanical ventilation

Supplementary Figure 1. BTT over time among LTx cases included in this study. (A) The number of BTT and non-BTT cases in each year from 2008 to 2021. (B)


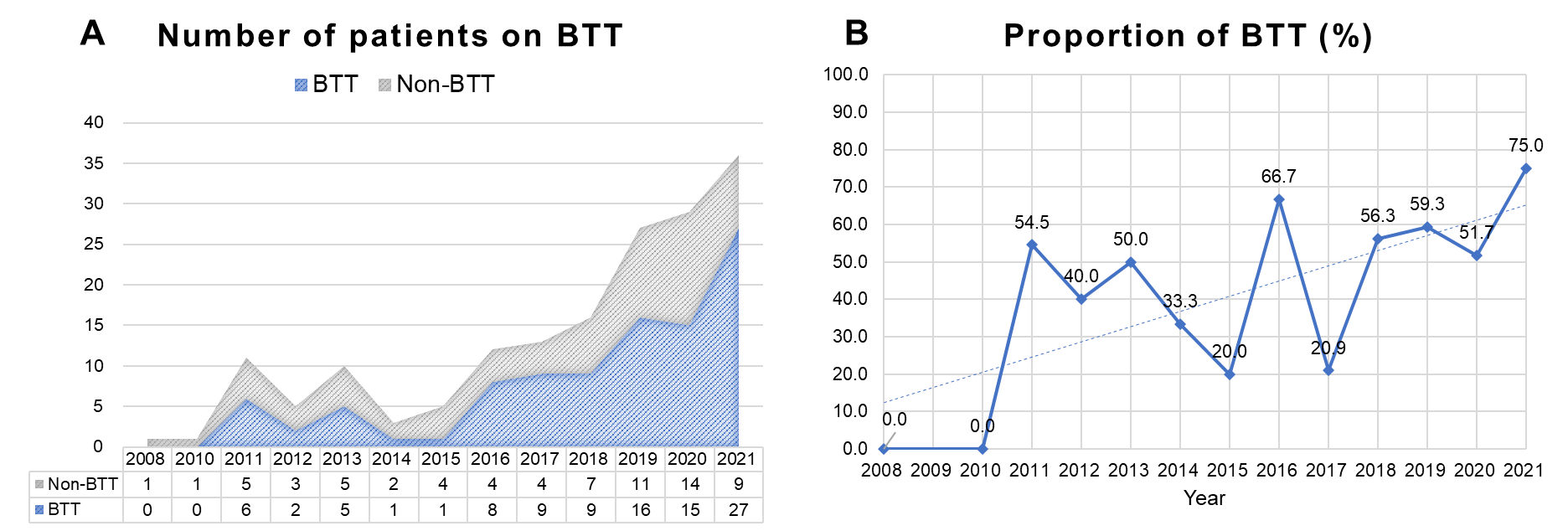


BTT; bridge-to-transplantation, LTx; lung transplantation

Supplementary Figure 2. Univariate analysis on risks for 28-day mortality. Hazard ratios (HR) and 95% confidence intervals (CI) are given for each variable.


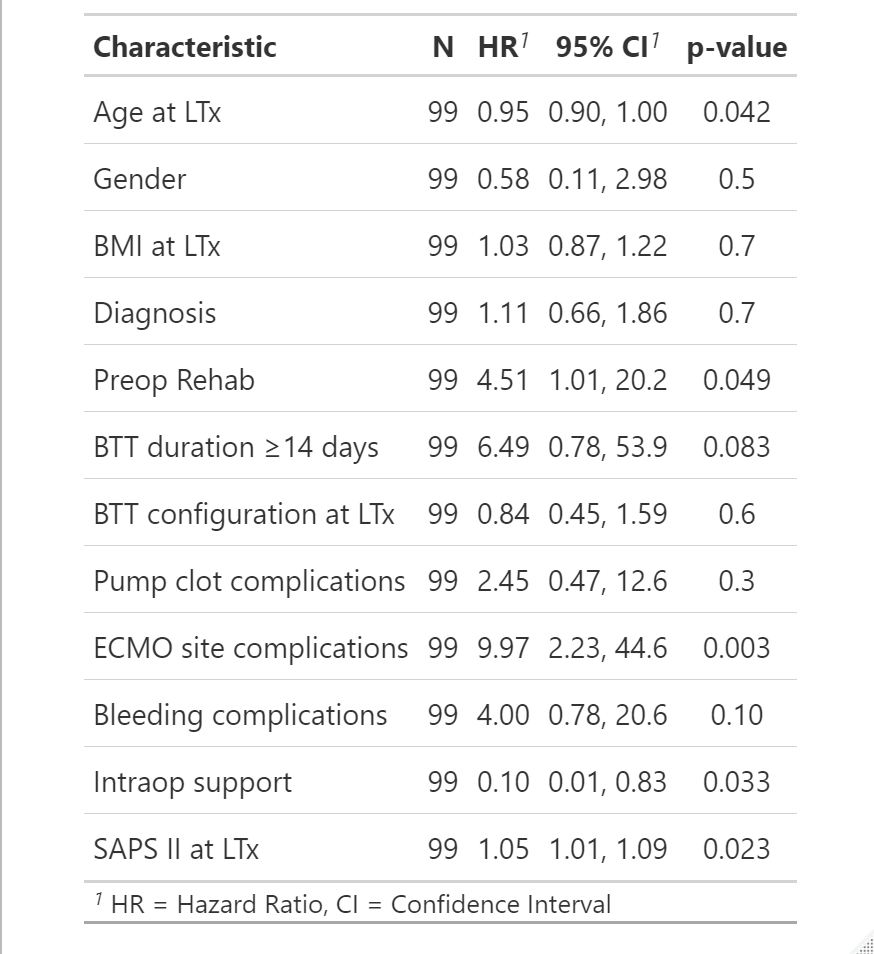


LTx; lung transplantation, BMI; body-mass index, preop; preoperative, BTT; bridge-to-transplantation, ECMO; extracorporeal membrane oxygenator, intraop; intraoperative, SAPS II; Simplified Acute Physiology Score II

Supplementary Figure 3. Multivariable analysis on risks for 28-day mortality using logistic regression. Hazard ratios and 95% confidence intervals are presented next to the forest plot.


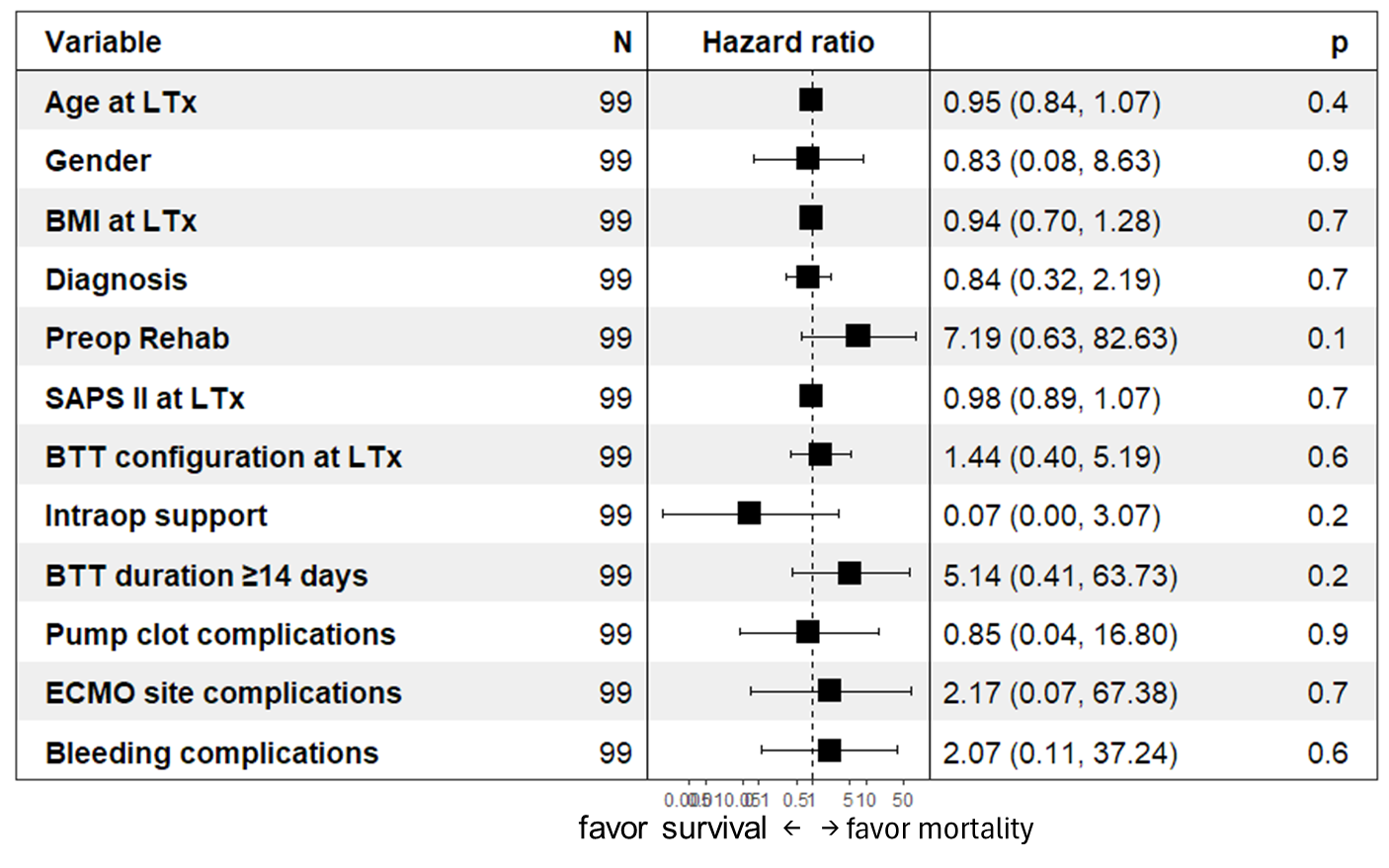


LTx; lung transplantation, BMI; body-mass index, BTT; bridge-to-transplantation, preop; preoperative, intraop; intraoperative, SAPS II; Simplified Acute Physiology Score II, ECMO; extracorporeal membrane oxygenator
